# Subjective happiness moderates the relationship between implicit and explicit attitudes and excessive digital media use among adolescents

**DOI:** 10.1038/s41598-026-43516-6

**Published:** 2026-03-10

**Authors:** Jakub Hladik, Karla Hrbackova, Anna Petr Safrankova

**Affiliations:** Faculty of Humanities, Tomas Bata University in Zlín, Štefánikova 5670, Zlín, 760 01 Czech Republic

**Keywords:** Subjective happiness, Adolescents, Implicit attitudes, Explicit attitudes, Self-control, Excessive digital media use, Health care, Psychology, Psychology

## Abstract

Excessive digital media use (EDMU) represents a growing mental health concern among adolescents. However, it is unclear how implicit and explicit attitudes—automatic emotional vs. conscious rational evaluation—contribute to EDMU, and how these mechanisms differ according to subjective happiness. We analyzed data from 1,425 adolescents (M age = 13.12), to compare high (*n* = 312) and low subjective happiness (*n* = 171). Unhappy adolescents exhibited higher EDMU and impulsivity, and lower self-control compared to their happier peers. In this group, positive implicit attitudes toward social media predicted EDMU both directly and indirectly through impulsivity and self-control, while explicit attitudes had only a direct effect. No significant effects of attitudes were found among happy adolescents; however, with higher self-control having a protective effect. The results suggest that implicit and explicit attitudes influence EDMU differently, with their impact depending on subjective happiness, highlighting the need for a graded approach in intervention strategies.

## Introduction

Digital technologies have become an integral part of adolescents’ everyday lives, with young people spending an increasing amount of time online^1^. This trend is particularly evident in relation to social media use, with adolescents exhibiting higher usage rates than adult users^2^. Between 2012 and 2018, for example, the percentage of adolescents using social media several times a day increased from 34% to 70%^3^. This increase is also reflected in the prevalence of problematic digital media users.

Digital media, particularly social media, have several positive effects such as facilitating social connections, enhancing well-being, and providing opportunities for self-presentation and social support^4–6^. However, the use of digital media is also associated with behaviours that can have negative consequences. In this context, the study uses the term excessive digital media use (EDMU), based on the concept of excessive Internet use^7^. EDMU is characterised by a failure to control the impulse to use digital media, often leading to using it for longer than intended and continuing despite adverse consequences^7,8^. EDMU is a continuum of digital media use, where the most serious end of the spectrum may include users who are at risk of developing symptoms indicative of addictive behaviour^9^. This nuanced approach is based on Griffiths’ definition^10^, which is used to assess the risk of behavioral addictions^11,12^. According to this framework, a behavior is classified as an addiction only if it meets all six core components (salience, mood modification, tolerance, withdrawal, conflict, and relapse) simultaneously, and involves negative consequences that affect an individual’s life. In contrast, the term EDMU is used to capture a broader spectrum of dysfunctional digital media use that may impair daily functioning and psychological well-being^13^, but does not meet the full symptomatic threshold set of criteria required for a clinical addiction. This distinction allows for the identification of different levels of problematic engagement without using the term ‘addiction’^7^.

In addition to the amount of time spent using digital technologies, the nature of their use and its negative consequences also play a significant role^14^. Research indicates that EDMU may be associated with physical factors (e.g., sleep problems and obesity), cognitive factors (e.g., executive functioning and academic performance) and emotional and behavioural factors^12,15–17^. Furthermore, studies have found an association between digital technology use and lower psychological well-being and happiness among adolescents^18–21^. For instance, although social media can facilitate positive interactions and connections, excessive use (particularly when it replaces face-to-face interactions) can lead to feelings of isolation and dissatisfaction (due to social comparison, for example)^22–24^. However, this relationship is often complex^25,26^.

Subjective happiness (SH) is a key component of the broader, multidimensional concept of subjective well-being (SWB). SWB is based on subjective evaluations of life and encompasses both affective experiences, namely the predominance of positive emotions, and a cognitive component, such as rational evaluations of life satisfaction^27^. In contrast to SWB as a whole, SH represents its narrower affective component, reflecting the subjective perception of the frequency and intensity of positive and negative emotions over a specific period of time^28,29^. This perspective is grounded in the hedonic conceptualization of SWB, which focuses on the maximization of positive affect and pleasurable experiences^30^.

Due to its affective and time-sensitive nature, SH is relevant to research on behaviors related to impulsivity and self-regulation, particularly in adolescents, a developmental period characterized by fluctuating affective states^29,31^. SH, which reflects an individual’s affective state, can influence self-regulatory processes and affect the choice between immediate gratification and long-term goals^32^. Negative emotional state may cause individuals to prioritize short-term mood regulation (“making themselves feel better”)^33^. Maladaptive use is more likely when psychosocial problems precede online activities^34^. This suggests that negative subjective states may directly impact motivation and strategic decisions when individuals believe these states are changeable^33^. In such cases, a failure of self-control may reflect not only limited regulatory capacity, but also a conscious or strategic re-evaluation of priorities, favoring immediate relief through DMU^33,35^. Conversely, individuals with higher levels of SH show a greater capacity for adaptive self-regulation and more effective use of coping strategies in problem solving^29^.

For this reason, the present study differentiates adolescents according to relatively higher versus lower levels of SH. This distinction allows for the identification of distinct mechanisms linking social media attitudes, self-control, and impulsive or excessive digital media use—mechanisms that broader and more stable constructs of well-being might not reveal. Given the unique developmental characteristics of adolescence, this approach offers a more nuanced and granular understanding of digital behavior.

Dual-process theories^36–38^ provide a framework for understanding the dynamics of digital media use. These theories assume the existence of two parallel systems. The impulsive system (1) is constantly active and influences behaviour through associative links and motivational orientations. It is characterised by a tendency to act on immediate impulses without considering the consequences^39^. The reflective system (2) is controlled and associated with conscious reasoning, long-term goals and values^40^. These two systems represent two different processing modes that may or may not interact with each other^37^. They correspond to what is known as ‘slow and fast thinking’^41^. The impulsive system reacts more quickly and automatically, whereas the reflective system requires more cognitive resources. In situations where strong impulsive motives prevail (e.g., the urge to respond immediately to a message), System 1 dominates System 2^8,40^. Consequently, behaviour is ultimately driven more by unconscious, impulsive motives than by deliberate self-control^36^.

Self-control plays a key role in this interaction. It is defined as an individual’s ability to change or inhibit their impulsive tendencies towards immediate gratification or change their dominant responses and prioritize goal-oriented behaviour^36,42,43^. Self-control enables the reflective system to inhibit behaviour that is activated impulsively and could conflict with values or long-term goals^36,44,45^. However, self-control requires a high level of mental resources, which may be limited. This may include cognitive resources (e.g., executive functions), as well as motivational resources (i.e., the energy, effort, and perceived value of the long-term goal required to exert deliberate control)^32,46–48^. When these resources are weakened or depleted, an individual’s ability to resist impulses related to digital media decreases^45,49,50^, thereby increasing the risk of excessive digital media use^51^. Impulsive reactions are not always associated with excessive or harmful behaviour. Impulsive behaviour becomes unhealthy when performed in an inappropriate context and the individual cannot control it^48,52^.

Zahrai et al.^8^ point out that, in addition to self-control, which is generally beneficial for healthy media use, individuals’ attitudes towards the media also significantly influence their behaviour. In this context, dual-system theories can be interpreted as models of dual attitudes^53^. Here, an attitude is defined as a psychological evaluation of an object with a certain degree of sympathy or antipathy. An individual may hold implicit and explicit attitudes towards the same object (e.g., social media) that are inconsistent with each other and influence behaviour differently^54^. Specifically, explicit attitudes are conscious, intentional evaluations that require cognitive abilities^54^. They are associated with the reflective system and influence conscious reactions to a given object. In contrast, implicit attitudes are subconscious, relatively stable evaluations activated with little or no conscious effort in response to internal or external stimuli^8,54^. They influence spontaneous, uncontrolled reactions and are closely linked to the impulsive system. Users are generally unaware of their implicit attitudes and how these influence their behaviour (e.g., automatically opening applications)^54,55^. This can be problematic when these attitudes promote impulsive digital media use. Research shows that implicit attitudes towards social media are a key predictor of impulsive use, more so than explicit attitudes. This discrepancy can disrupt the harmony between declared intentions (e.g., limiting time online) and actual behaviour, which is often automated and impulsive. Although self-control is considered a protective factor, it may not be sufficient for individuals with positive implicit attitudes and high impulsivity Furthermore, research demonstrates that implicit attitudes exert significant indirect effects on behavioral outcomes, primarily through the mediation of impulsive media use^8^.

Together, dual-process theory, dual-attitude theory, and self-control models provide complementary perspectives on the mechanisms underlying media-related behavior. Dual-process theory distinguishes between reflective and automatic pathways of behavior regulation, while dual-attitude theory specifies that explicit and implicit attitudes can coexist and shape tendencies toward a given behavior. Self-control models further explain how individual differences in regulatory capacity influence which pathway is more likely to guide behavior in a given situation. Within this process, behavior is viewed as the result of a dynamic interaction between these pathways. Rather than representing competing accounts, these frameworks address different levels of explanation. In the present study, we integrate these perspectives into a unified conceptual model.

In summary, extant research among adult samples highlights the role of self-control and social media attitudes in digital behaviour through the so-called ‘duality of self-control’, whereby cognitive control ceases to be an effective regulator when implicit attitudes toward the media are strongly positive and impulsivity is high^8^. These mechanisms, however, have not been comprehensively examined in adolescents, a developmental period characterized by the asynchronous maturation of two systems, where the socioemotional system (motivation, rewards) matures earlier than the cognitive control (self-control). It is precisely the time lag between the maturation of the socio-emotional system and the cognitive control system that may represent a period of increased vulnerability to excessive digital media use^31^. While SH is often conceptualized as a predictor or mediator in adult populations^56^, divergent dynamics may be anticipated in adolescents due to the aforementioned developmental specificities. Drawing upon the theory of digitally regulated emotions^57^, adolescents utilize digital media as a mechanism to regulate affective states originating in offline environments. In this context, it remains unclear how SH moderates the underlying pathways within dual-process models through which attitudes translate into excessive digital media use among adolescents. Understanding this dynamic is therefore essential for elucidating the mechanisms leading to EDMU and for designing effective preventive interventions for adolescents.

## Research objectives and hypotheses

Following the theoretical framework outlined above, this study pursues two main objectives: first, to investigate differences in attitudes toward social media (both implicit and explicit), impulsive digital media use, self-control, and excessive media use between happy and unhappy students (Objective 1); and second, to examine the direct and indirect effects of attitudes toward social media on excessive media use (Objective 2). Based on these objectives, we formulated the following hypotheses:

### Hypothesis 1

(H1): The mechanisms through which implicit and explicit attitudes toward social media influence excessive digital media use differ between happy and unhappy students.

### Hypothesis 2

(H2): Among unhappy students, both implicit and explicit attitudes toward social media will be significantly associated with excessive digital media use.

For this purpose, we designed four models to represent the hierarchical structure of relationships among implicit and explicit attitudes toward social media, impulsive digital media use, self-control, and excessive digital media use. To demonstrate the effects of either implicit or explicit attitudes, two models are presented with either implicit or explicit attitudes as predictors. Additionally, the models are presented separately for happy and unhappy students to facilitate comparison.

## Methods

### Participants

Participants (*N* = 1,425; 704 girls, 714 boys, seven unreported) were lower-secondary school students from randomly selected primary schools in the Czech Republic. A stratified random sampling procedure was employed to select participating schools. The sampling frame consisted of a complete registry of primary schools provided by the Ministry of Education, Youth and Sports of the Czech Republic. Schools were stratified by region (NUTS 3 level) to ensure proportional geographic representation. From this registry, schools were randomly invited to participate. The mean age was 13.12 years (SD = 1.17; range = 11–16 years), covering early to mid‑adolescence. Research indicates that adolescents in this age range may be especially susceptible to impulsive and excessive engagement with digital media, as immature self‑regulatory processes are associated with difficulties in resisting rewarding stimuli such as social media and online content^58^.To test our hypotheses under conditions of maximum theoretical contrast, participants were divided into two groups based on their subjective happiness scores. This categorical approach was chosen to isolate the mechanisms of self-regulation in clearly distinct psychological states, consistent with a theory-driven extreme group design strategy^59^. For our research, we selected a subsample of 312 happy students (134 girls, 178 boys; M = 13.03 years; SD = 1.13; age range = 5) and 171 unhappy students (117 girls, 53 boys, one unreported; M = 13.28 years; SD = 1.27; age range = 5) from the total sample. This selection was based on a single questionnaire item asking, “How have you felt over the last six months?” Participants rated their feelings on an 11-point scale ranging from “very unhappy” to “very happy”. We identified students with responses ranging from 0 to 3 as unhappy (M = 2.06, SD = 0.99) and students with responses from 8 to 10 as happy (M = 8.76, SD = 0.78). The division of the scale into 0–3 and 8–10 intervals corresponds to the extreme poles of subjective experience – low versus high levels of happiness – which is a common interpretation for 0–10 scales in psychological and sociological research^60^.

The research was conducted by teachers who voluntarily agreed to administer the study during a single lesson period (45 min). Teachers were instructed to administer the research study under standardized conditions, ensuring that students completed all questionnaires individually and without distraction on an online platform. The teachers’ role was limited to supervision, technical assistance, and maintaining the standardized time frame. No financial compensation was provided to the teachers for their participation. Participants completed all questionnaires on an online platform. The research was conducted with the informed consent of the participants’ legal guardians. Although each participant could opt to decline participation or withdraw from the study at any time, no one did. The research was approved by the Ethics Committee of Tomas Bata University on March 23, 2023 and conducted in accordance with relevant guidelines and the Declaration of Helsinki.

### Measures

#### Implicit attitude toward social media

To measure implicit attitudes towards social media, we adapted the Single Category Implicit Association Test (SC-IAT)^61^. The SC-IAT was employed, justified by its favorable psychometric characteristics^61,62^ and its unique capacity to assess the strength of mental associations with a single attitude object lacking an obvious opposite category. We developed word sets across three categories: negative words, positive words, and online world-related words. The negative and the positive word categories comprised ten terms each (e.g., bad, boring, annoying, and good, easy, entertaining, respectively). The online world-related category included five terms: digital/online games, internet, smartphone, videos/YouTube/TikTok, and social media.

The test was administered on a computer. The upper left corner of the screen displayed a designated area for negative words, while the upper right corner was designated for positive words. Participants were presented with negative or positive words in random order on the screen and instructed to categorize each word as quickly as possible using the “E” button for negative words and the “I” button for positive words on the keyboard. In subsequent blocks, words related to the online world were introduced. These were presented alongside either the negative words (on the left side of the screen) or the positive words (on the right side). Participants were consistently instructed to make their choices as swiftly as possible. In the event of an error, an error message appeared on the screen, prompting participants to self-correct.

The entire SC-IAT was divided into five blocks. The first block served as a practice phase, requiring participants to categorize a set of ten negative and ten positive words twice. In the second block, also a practice phase, participants matched words related to the online world with positive words. The third block was a test block during which participants’ reaction times were measured while they assigned words. In this block, they matched negative words (displayed in the left corner) with positive words and words related to the online world (displayed in the right corner). This entire set was repeated twice. The fourth block was another practice phase, similar to the second block, except that the participants had to match words related to the online world with negative words. The fifth block was a test block, again involving reaction time measurements, where participants matched negative words and online world-related words (in the left corner) with positive words (in the right corner).

The scoring is based on the principle that participants with a positive implicit attitude are quicker to match words related to the online world with positive words, and vice versa for a negative implicit attitude. Only two measured blocks were scored^61,63^. Responses with reaction times shorter than 350 ms were excluded. A penalty of 400 ms was added to the reaction time for incorrect responses. The D-score, indicating implicit attitude, was obtained by subtracting the average response time of the fifth block (associated with negative words) from that of the third block (associated with positive words), and then dividing this difference by the standard deviation. For this article, we will focus solely on implicit attitudes toward social media, which was one of the five terms related to the online world included in the SC-IAT. A D-score of less than zero indicates a positive implicit attitude toward social media, while a score greater than zero signifies a negative implicit attitude. The magnitude of the score, as its distance from zero, reflects the strength of this association.

In the present study, the internal consistency of the SC-IAT was assessed using the Spearman-Brown corrected split-half reliability, yielding a coefficient of 0.41. While this value is relatively modest in comparison with traditional self-report measures, it is consistent with the psychometric properties observed in single-category implicit association tasks, where reliability coefficients are typically lower, particularly within adolescent samples^64^.

#### Explicit attitude toward social media

We developed a 10-item tool based on semantic differential principles for measuring explicit attitudes toward social media. The following pairs of bipolar adjectives were used: good – bad; pleasant – unpleasant; friendly – unfriendly; entertaining – boring; positive – negative; relaxed – tense; beneficial – pointless; comforting – hurtful; delightful – annoying; problematic – unproblematic. Based on the exploratory factor analysis results, we decided on a single-factor solution measuring attitudes towards social media. The data do not strictly adhere to the normality assumption; therefore, Principal Axis Factoring was used for factor extraction, which is considered robust against this violation^65^. The factor loading was from 0.52 to 0.74. KMO test = 0.872. Bartlett’s test *p* < 0.001. The single-factor model explains 48% of the variance of items. Confirmatory factor analysis (CFA) was used to test the fit of the model. The CFA results are satisfactory (*p* = 0.004; TLI = 0.939; CFI = 0.969; RMR = 0.097; RMSEA = 0.078; PCLOSE = 0.004). Although the p-value is less than 0.05, the good values of the fit indices, the strong item-to-item correlations, and the higher factor loadings collectively support our chosen single-factor solution. The Cronbach’s alpha was 0.88.

#### Impulsive digital media use

Our research adapted items from the Compulsive Internet Use Scale (CIUS)^66^ to measure impulsive digital media use. We exclusively utilized items related explicitly to impulsive digital media use: “I often continue to use social media even though I intend to stop”; “I find it difficult to stop using the internet when I am online “; “I have been told many times that I spend too much time on my smart phone”; “I stay on my smart phone longer than I intended” and “I am impatient and rush through assignments to get online”. These five items were measured on a five-point scale ranging from “all” to “still.” CIUS is used in several studies^67,68^. The Cronbach’s alpha was 0.76.

#### Self-control

Students’ self-control was assessed using *The Brief Self-Control Scale*^69^. This 13-item instrument, scored on a five-point Likert scale (from “not at all” to “very much”), examines various behavioral facets of self-control, such as achievement, impulse regulation, psychological well-being, interpersonal interactions, moral affect, and personality. This scale is often used to assess the level of self-control^44,70,71^. The Cronbach’s alpha was 0.75.

#### Excessive digital media use

In our research, we use the term “digital media,” which encompasses smartphones, computers, and more generally, internet access on these devices. Students’ self-reported internet use over the prior six months was assessed using the Excessive Internet Use Scale^9^. Drawing from Griffiths’^10^ model of behavioral addictions, this five-item tool captures sustained patterns of internet engagement rather than short-term habits. The scale’s items address key aspects of problematic use, such as salience (“I have gone without eating and sleeping because of the internet”), withdrawal symptoms (“I have felt bothered when I cannot be on the internet”), tolerance (“I have caught myself surfing when I am not really”), relapse (“I have tried unsuccessfully to spend less time on the internet”), and conflict (“I have spent less time than I should with either family, friends, or doing schoolwork because of the time I spend on the internet”). We utilized a five-point rating system instead of the original four-point format to achieve consistency with other scales in our research. This tool is used by scholars to determine the extent of internet excessiveness^72,73^. The Cronbach’s alpha was 0.71.

We conceptualize digital media use as a broad behavioral domain encompassing engagement with internet-based and platform-mediated activities. This domain includes several media genres: social media use, general internet use, entertainment-oriented online activities (e.g., gaming, streaming), and other forms of online interaction. While these constructs represent distinct behavioral contexts, they are theoretically related through shared underlying mechanisms of self-regulation and behavioral control. To capture this complexity, we operationalize our primary constructs using specific facets of this domain: implicit and explicit attitudes were assessed regarding social media, as these platforms represent the most affectively salient digital environment for adolescents, while excessive behavior was measured via generalized problematic internet use. This approach allows us to study multiple relevant aspects of digital media behavior without conflating conceptually distinct phenomena, providing a nuanced understanding of how attitudes toward a specific genre (social media) relate to broader problematic patterns across the digital sphere.

### Analysis

Analysis of normality using the Shapiro-Wilk test revealed that several variables were not normally distributed. For the happy student group, these variables were implicit attitude toward social media, explicit attitude toward social media, impulsive digital media use, and excessive digital media use. For the unhappy group, the data were not normally distributed for implicit attitude toward social media, explicit attitude toward social media, self-control, and excessive digital media use. Given these deviations from normality, we used bootstrapping to obtain robust standard errors and confidence intervals for all parameter estimates^74,75^. Confirmatory factor analysis was conducted to test the validity of the single-factor solution for the explicit attitude scale. Bootstrapping with 5,000 resamples was used for robust estimation. An independent-samples t-test was performed to compare the two participant groups of happy vs. unhappy students. Bootstrapped confidence intervals for the mean difference were estimated using 10,000 bootstrap samples. Preliminary analyses (t-tests) were conducted to explore potential gender differences. No significant differences were found between boys and girls in excessive digital media use, implicit attitudes, or self-regulatory variables; only a minor difference was observed in explicit attitudes, with boys reporting slightly more positive evaluations. Consequently, gender was not included in the final path models to ensure model parsimony.

To ensure conceptual rigor and a nuanced comparison, we adopted a quadripartite analytical framework comprising four distinct models. This design was chosen for two primary reasons. First, it allows for a systematic comparison between implicit and explicit cognitive systems, preventing statistical overlap and suppression effects that often occur when these constructs are entered simultaneously into a single model. Second, by analyzing happy and unhappy adolescents separately, we can pinpoint specific self-regulatory patterns that may be unique to each group.

To examine the potential moderating role of happiness on the relationship between attitudes toward digital media use and excessive digital media use, we conducted moderation analyses using the PROCESS macro for SPSS (version 4.2)^76^. For both explicit and implicit attitudes, we applied Model 1 with subjective happiness as the moderator. All predictor and moderator variables were mean-centered prior to analysis, and heteroscedasticity-consistent standard errors (HC3) were used. The Johnson–Neyman technique was applied to identify regions of the moderator where the conditional effect of attitudes was statistically significant, thereby empirically informing the interpretation of “happy” versus “unhappy” adolescents. To further examine the full mediation pathway including self-control (SC) and impulsive digital media use (IDMU) as mediators, we conducted Model 85, which allowed testing whether happiness moderates both the direct effect of attitudes on excessive digital media use (EDMU) and the indirect effects via SC and IDMU. Conditional direct and indirect effects were estimated at the 16th, 50th, and 84th percentiles of happiness to illustrate the moderating effect across low, medium, and high levels of the moderator. Bootstrapping with 5,000 resamples was used to obtain robust confidence intervals for all parameter estimates. Finally, we estimated four multi-group structural path models to test the hypothesized mechanisms. Specifically, two path models were estimated for adolescents with low subjective happiness and two path models were estimated for those with high subjective happiness. In each group, one model included implicit attitudes as the exogenous predictor, and the other included explicit attitudes as the exogenous predictor. Although we describe certain variables as ‘predictors’ or ‘predicted’ in these models, this terminology refers to statistical associations estimated in the path analysis and does not imply causal relationships, given the cross-sectional design of the study. Nevertheless, these associations may indicate potential pathways linking attitudes, self-regulation, and excessive digital media use, highlighting possible interpretations without implying definitive causality. This approach allowed us to compare the indirect effects and test whether the mechanisms linking attitudes to excessive digital media use differed between groups and enabled us to identify the moderating effect of subjective happiness. Robust standard errors and confidence intervals for the path coefficients were calculated using 5,000 bootstrap samples.

Cohen’s *d* was used alongside the t-test to indicate the effect size between happy and unhappy students. According to Cohen^77^, values greater than 0.80 indicate a large and practically significant difference between groups. The fit indices reported in path analysis are typically used to evaluate the quality of the model^78^.

All analyses were conducted in IBM SPSS Statistics 29.0 and IBM SPSS Amos 29.0 with the significance level set at α = 0.05.

## Results

### Differences in attitudes, social media use, and self-control between happy and unhappy students

Table 1 shows the implicit attitude toward social media of unhappy and happy students. Negative index values indicate a positive implicit attitude toward social media, which is valid for both groups. The greater the distance of the value from zero, the more positive the implicit attitude toward social media. However, it is shown that this difference in implicit attitude toward social media between happy and unhappy students is not statistically significant (*p* = 0.275). This is the only variable for which there is no statistically significant difference between happy and unhappy students. Happy students have a more statistically significant positive explicit attitude toward social media (M_expl_ = 4.40; SD_expl_ = 1.07) than unhappy students (M_expl_ = 4.20; SD_expl_ = 1.02; *p* = 0.042). Even though happy students have more positive explicit attitude toward social media, they have lower excessive digital media use (M_exc_ = 1.75; SD_exc_ = 0.62) than unhappy students (M_exc_ = 2.32; SD_exc_ = 0.85). In addition, unhappy students have higher impulsivity of digital media use (M_impu_ = 2.77; SD_impu_ = 0.93) and lower self-control (M_sc_ = 2.51; SD_sc_ = 0.84) than happy students (M_impu_ = 2.26; SD_impu_ = 0.82; M_sc_ = 3.29; SD_sc_ = 0.81). These differences are statistically significant (*p* < 0.001). Moreover, the differences in explicit attitude, impulsive digital media use, self-control, and excessive digital media use are large between happy and unhappy students according to Cohen’s *d*. For explicit attitudes, the effect on excessive digital media use was significant only among adolescents with low happiness (b = −0.080, *p* < 0.001), whereas for high happiness, the effect was non-significant (b = 0.008, *p* = 0.233). For implicit attitudes, the effect was negative and significant at low happiness (b = −0.066, *p* = 0.035) and positive and significant at high happiness (b = 0.073, *p* = 0.006), whereas effects at moderate levels of happiness were non-significant. These results indicate that the moderating role of subjective happiness emerges primarily at the extremes of the distribution. Therefore, describing the results in terms of “happy” versus “unhappy” adolescents is justified, as it reflects the participants for whom the effect of attitudes on excessive digital media use is practically and statistically meaningful. In the full moderated mediation model, the direct and indirect effects of attitudes on excessive digital media use via self-control (SC) and impulsive digital media use (IDMU) were similarly moderated by happiness at the extremes, confirming the robustness of the moderating effect across the full model. For instance, for implicit attitudes, low happiness was associated with a conditional direct effect on EDMU of b = −0.066 (*p* = 0.035), and high happiness with b = 0.073 (*p* = 0.006). For explicit attitudes, low happiness yielded a direct effect of b = −0.080 (*p* < 0.001), whereas high happiness was non-significant (b = 0.008, *p* = 0.233).

**Table 1:** Differences in attitudes, social media use, and self-control between happy and unhappy students.

Variable	Unhappy students		Happy students		t (481)	*p*	Cohen’s d
	M	SD	M	SD			
Implicit attitude toward social media	− 0.09	0.96	− 0.18	0.87	1.13	0.275	0.90
Explicit attitude toward social media	4.20	1.02	4.40	1.07	−2.00	**0.042**	1.05
Impulsive digital media use	2.77	0.93	2.26	0.82	6.17	**< 0.001**	0.86
Self-control	2.51	0.84	3.29	0.81	−10.02	**< 0.001**	0.82
Excessive digital media use	2.32	0.85	1.75	0.62	8.43	**< 0.001**	0.80

### Models of the influence of implicit and explicit attitudes on excessive digital media use

The figures show the influence of implicit and explicit attitudes on the excessive digital media use through impulsivity and self-control, for both happy and unhappy students. Table 2 shows the path models fit indices. The excellent values of the fit indices in the path models indicate that the proposed models fit the observed data very well. The direct and indirect effects of each variable in the models are reported in Tables 3 and 4.

**Table 2:** Path models fit indices.

Model	CMIN	CMIN/DF	bootstrap *p*-value	CFI	TLI	RMSEA	PCLOSE	RMR
Unhappy students								
The influence of implicit attitude on excessiveness	0.329	0.329	0.504	1.0	1.0	0.001	0.641	0.009
The influence of explicit attitude on excessiveness	3.746	3.746	0.123	0.984	0.903	0.127	0.104	0.033
Happy students								
The influence of implicit attitude on excessiveness	1.983	0.991	0.366	1.0	1.0	0.001	0.606	0.015
The influence of explicit attitude on excessiveness	0.206	0.103	0.920	1.0	1.0	0.001	0.953	0.004

Model 1 estimated both direct (β = − 0.15) and indirect effects of implicit attitude on excessive digital media use via impulsivity and self-control. For unhappy students, the indirect effect of implicit attitudes on excessiveness (see IA → EDMU for Model 1 in Table 4) is weaker (β = − 0.08) than the direct effect. The more positive the students’ implicit and explicit attitude is, the higher their excessive digital media use will be. All relationships in Model 1 are statistically significant, as shown by the p-values in Tables 3 and 4. **The implicit attitude of unhappy students plays a significant role in the excessive digital media use.**

**Fig. 1:**
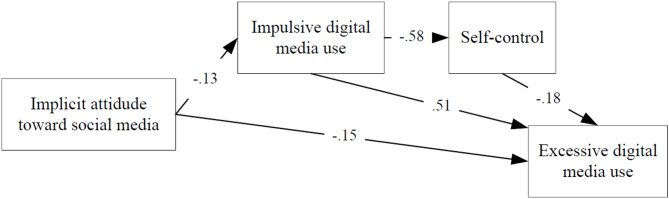
Path diagram of the effect of implicit attitude on excessive digital media use in unhappy students (Model 1).

**Table 3:** Path models of direct effects.

	The influence of implicit attitudes on excessive digital media use									
	Unhappy students (Model 1)					Happy students (Model 2)				
				95% CI					95% CI	
Direct Paths	b	*p*	β	LB	UB	b	*p*	β	LB	UB
IA → IDMU	**− 0.123**	**0.045**	**− 0.128**	**− 0.251**	**− 0.005**	− 0.015	0.802	− 0.016	− 0.114	0.094
IDMU → SC	**− 0.527**	**<0.001**	**− 0.580**	**− 0.630**	**− 0.430**	**− 0.567**	**0.001**	**− 0.571**	**− 0.663**	**− 0.467**
SC → EDMU	**− 0.185**	**0.008**	**− 0.184**	**− 0.319**	**− 0.044**	**− 0.207**	**<0.001**	**− 0.271**	**− 0.306**	**− 0.109**
IDMU → EDMU	**0.463**	**<0.001**	**0.505**	**0.309**	**0.611**	**0.303**	**<0.001**	**0.398**	**0.191**	**0.412**
IA → EDMU	**− 0.129**	**0.012**	**− 0.146**	**− 0.223**	**−030**	-	-	-	-	-

	The influence of explicit attitudes on excessive digital media use									
	Unhappy students (Model 3)					Happy students (Model 4)				
				95% CI					95% CI	
	b	*p*	β	LB	UB	b	*p*	β	LB	UB
EA → IDMU	0.130	0.092	0.143	− 0.025	0.278	0.075	0.123	0.099	− 0.025	0.219
IDMU → SC	**− 0.527**	**<0.001**	**− 0.580**	**− 0.630**	**− 0.430**	**− 0.567**	**<0.001**	**− 0.571**	**− 0.648**	**− 0.488**
SC → EDMU	**− 0.205**	**0.005**	**− 0.203**	**− 0.341**	**− 0.067**	**− 0.207**	**<0.001**	**− 0.271**	**− 0.393**	**− 0.142**
IDMU → EDMU	**0.489**	**<0.001**	**0.533**	**0.343**	**0.631**	**0.303**	**<0.001**	**0.398**	**0.259**	**0.531**
EA → EDMU	**− 0.127**	**0.005**	**− 0.152**	**− 0.211**	**− 0.043**	-	-	-	-	-

IA = implicit attitude; IDMU = impulsive digital media use; SC = self-control; EA = explicit attitude; EDMU = excessive digital media use; *p* = bootstrap p-value; LB = lower bounds; UB = upper bounds; CI = 95% confidence interval.

**Table 4:** Path models of indirect effects.

	The influence of implicit attitudes on excessive digital media use									
	Unhappy students (Model 1)					Happy students (Model 2)				
				95% CI					95% CI	
Indirect paths	b	*p*	β	LB	UB	b	*p*	β	LB	UB
IA → SC	**0.065**	**0.044**	**0.074**	**0.003**	**0.147**	0.008	0.805	0.009	− 0.057	0.067
IA → EDMU	**− 0.069**	**0.042**	**− 0.078**	**− 0.151**	**− 0.004**	− 0.006	0.812	− 0.009	− 0.067	0.055
IDMU → EDMU	**0.098**	**0.007**	**0.098**	**0.028**	**0.190**	**0.188**	**<0.001**	**0.155**	**0.083**	**0.227**

	The influence of explicit attitudes on excessive digital media use									
	Unhappy students (Model 3)					Happy students (Model 4)				
				95% CI					95% CI	
	b	*p*	β	LB	UB	b	*p*	β	LB	UB
EA → SC	− 0.069	0.085	− 0.083	− 0.178	0.014	− 0.043	0.114	− 0.056	− 0.126	0.013
EA → EDMU	0.078	0.085	0.093	− 0.016	0.200	0.032	0.117	0.054	− 0.013	0.126
IDMU → EDMU	**0.108**	**0.004**	**0.118**	**0.040**	**0.203**	**0.118**	**<0.001**	**0.155**	**0.083**	**0.227**

IA = implicit attitude; IDMU = impulsive digital media use; SC = self-control; EA = explicit attitude; EDMU = excessive digital media use; *p* = bootstrap p-value; LB = lower bounds; UB = upper bounds; CI = 95% confidence interval.

**Fig. 2:**
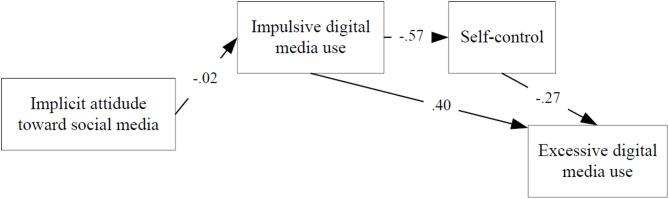
Path diagram of the effect of implicit attitude on excessive digital media use in happy students (Model 2).

Model 2 shows that, for happy students, implicit attitude has only an indirect effect on excessiveness. In the modelling process, when we considered a model in which there is also a direct effect on excessiveness, the model fit became worse. Indirect effect of implicit attitude on excessiveness is not strong (β = − 0.01) and it is not even statistically significant (see IA → EDMU for Model 2 in Table 4). Impulsive digital media use and self-control may influence excessive digital media use. Their direct and indirect effects have been found to be strong and statistically significant. However, **implicit attitude does not appear to play a significant role in this relationship in happy students**.

**Fig. 3:**
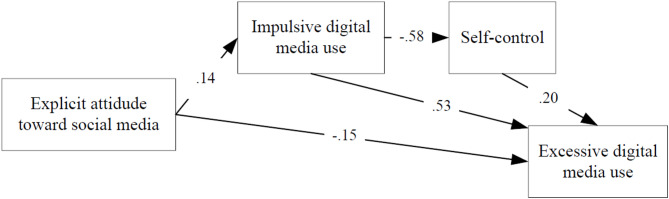
Path diagram of the effect of explicit attitude on excessive digital media use in unhappy students (Model 3).

Model 3 concerns unhappy students and shows possible ways of influencing excessiveness through explicit attitude. The direct effect of explicit attitude (β = − 0.15) on excessive digital media use is statistically significant (*p* = 0.005). The more positive the students’ explicit attitude is, the higher their excessive digital media use will be. Indirect effect of explicit attitude on excessiveness through impulsive digital media use and self-control is not statistically significant (see EA → EDMU for Model 3 in Table 4). **While implicit attitude affects excessive digital media use in unhappy students both directly and indirectly through impulsivity and self-control**,** explicit attitude affects it only directly.**

**Fig. 4:**
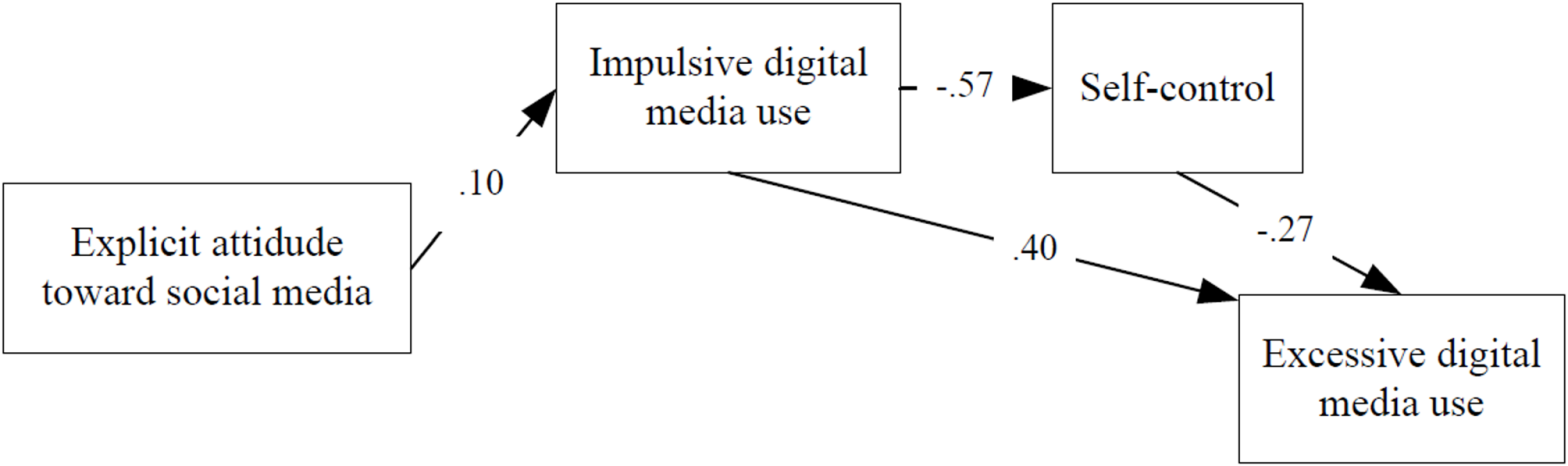
Path diagram of the effect of explicit attitude on excessive digital media use in happy students (Model 4).

As in model 2, in model 4, the direct effect of explicit attitude on excessiveness was removed in the modeling process to improve the fit of the model. The explicit attitude toward social media does not have a direct effect on excessive digital media use. However, the indirect effect of explicit attitude on excessiveness is not statistically significant (see EA → EDMU for Model 4 in Table 4). **Explicit attitude toward social media in happy students**,** as well as implicit attitude**,** does not influence excessive digital media use.**

## Discussion

The findings provide new insights into how implicit and explicit attitudes translate into behavior via different pathways depending on adolescents’ levels of subjective happiness. These findings contribute to the growing body of research exploring the psychological drivers of problematic digital media use in adolescence^79,80^. In line with dual models of self-regulation^36^, we assumed different pathways for reflective (explicit) and automatic (implicit) processes. The results confirm five main findings, which we incorporate into current research below and discuss their theoretical and practical significance (Figs. 1, 2, 3 and 4).

### Marked differences between happy and unhappy adolescents

The results showed consistent and statistically significant differences between happy and unhappy adolescents in almost all observed variables, with the exception of implicit attitudes. Unhappy adolescents exhibited higher levels of both impulsive and excessive digital media use, as well as lower levels of self-control, compared to their happy peers. This profile suggests that lower subjective happiness may be associated with a reduced ability to regulate digital behavior, which is consistent with previous research emphasizing the close relationship between emotional experience, self-regulation, and problematic use of digital media^20,21,81^. Although happy adolescents expressed more positive explicit attitudes toward social media, their actual behavior was less excessive. In contrast, unhappy adolescents rated social media less positively than happy adolescents, yet used it more impulsively and more frequently. This discrepancy between declared attitudes and behavior may reflect growing ambivalence – unhappy adolescents may have experienced negative consequences of excessive use (e.g., time loss, stress, conflicts), which may be reflected in their conscious attitudes^82^. Although their overall assessment of social media remains predominantly positive, it is less clear-cut than that of their happy peers. Unlike explicit attitudes, no differences were found between the groups in implicit attitudes. In both groups, implicit associations with social media were predominantly positive, suggesting that at the level of automatic processing, social media are perceived by adolescents as attractive, enjoyable, and emotionally engaging^83,84^. These implicit attitudes likely reflect broader cultural and developmental influences, such as the ubiquity of digital technologies, their central role in peers’ social lives, and their importance for self-presentation and identity^85,86^.

### Explicit positive attitudes toward social media predict excessive use only in unhappy adolescents

While both subjectively happy and unhappy adolescents generally expressed rather positive explicit attitudes toward social media, these attitudes played a different role in predicting excessive digital behavior. Among unhappy adolescents, more positive explicit attitudes toward social media were significantly associated with higher levels of excessive digital media use. This result may reflect a compensatory or escapist perception, whereby social media are not only perceived positively but also as necessary or emotionally satisfying. This interpretation is consistent with the compensatory internet use model^34^, which suggests that digital engagement can serve to alleviate negative affective states such as loneliness or boredom. Adolescents with low subjective happiness may be particularly vulnerable to this type of coping behavior, and their positive attitudes may reinforce use even when it becomes excessive or maladaptive.

In contrast, among subjectively happy adolescents, explicit positive attitudes toward social media were not associated with excessive use. These individuals use digital media in ways that are not driven by the compensation of unmet psychological needs, but rather with greater self-determination and regulation aligned with situational goals and the availability of offline alternatives. This difference illustrates how similar (positive) explicit attitudes can lead to different behavioral patterns depending on an individual’s emotional state^87,88^.

Unlike implicit attitudes, which influence behavior primarily through automatic pathways, explicit attitudes represent conscious, deliberately formulated evaluations. In our study, explicit attitudes were statistically significant predictors of excessive digital media use only in unhappy adolescents, and exclusively through a direct pathway, without mediation by impulsivity or self-control. This direct relationship may reflect the fact that positive explicit evaluations of media (e.g., as “pleasant” or “calming”) serve as a rationalization of behavior in emotionally vulnerable individuals. In other words, these attitudes may provide a cognitive framework that legitimizes frequent and intense media use, even when it has negative consequences. Explicit attitudes can thus function as a direct motivational force, leading to the targeted search for positive affective experiences or escape from negative emotions^82^.

### Implicit attitudes predict excessive digital media use primarily in unhappy adolescents

The results further showed that implicit attitudes toward social media significantly predict excessive digital media use, but only among unhappy adolescents. In this group, both direct and indirect associations emerged, with the indirect effect mediated by increased impulsivity and reduced self-control. This pattern aligns with dual-process models of self-regulation^36^, which distinguish between automatic (impulsive) and reflective (deliberate) processes of behavior control. Implicit attitudes, operating at an unconscious level, may thus trigger digital behaviors in emotionally vulnerable individuals beyond the scope of conscious control or deliberate evaluation.

While explicit attitudes influenced excessive digital media use in unhappy adolescents in a direct, conscious way, implicit attitudes had both direct and indirect effects, through weakened self-control and increased impulsivity. In line with the research of Cho & Kim^89^, it can be assumed that common digital cues such as notifications, app icons, or automated habits of media use act as strong “triggers” that activate impulsive responses. These reactions tend to be beyond the reach of the reflective system and are more likely to occur in situations of emotional dysregulation or reduced cognitive capacity (e.g., during fatigue, stress, or frustration)^82^. The research thus suggests that implicit attitudes are not just a passive reflection of a specific environment, but an active mechanism that, under certain conditions – especially in emotionally vulnerable individuals – can contribute to the emergence or maintenance of problematic digital behavior.

### Impulsivity and self-control as core mediators of digital media use

Regardless of the level of subjective happiness, impulsivity and self-control proved to be strong and consistent predictors of excessive digital media use (EDMU) across all models. These mediation pathways refer to statistical associations estimated in the path models and do not imply direct causal mechanisms. These self-regulatory mechanisms not only had significant direct effects on the excessive use but also mediated the relationship between attitudes – especially implicit ones – and actual behavior. As mediators, they played a key role, especially for implicit attitudes within the group of unhappy adolescents. For explicit attitudes, on the other hand, the mediation through impulsivity and self-control was not significant, suggesting that their influence on excessive use is more direct and statistically less dependent on these self-regulatory processes.

This finding confirms the central role of self-regulation in digital media use, as noted by Tangney et al.^69^, Hofmann et al.^87^, and LaRose et al.^90^. The strong negative relationship between self-control and EDMU was particularly significant. Adolescents with higher levels of self-control were significantly less likely to engage in excessive digital involvement, regardless of their attitudes or subjective happiness. Self-control thus represents an important psychological resource that enables individuals to resist impulsive tendencies and regulate behavior in accordance with long-term goals^45^. Its importance has been repeatedly confirmed in the context of coping with various forms of addictive and maladaptive behavior, including problematic technology use^43,91^.

In addition, impulsivity emerged as a significant predictor of EDMU and simultaneously as a partial mediator in the association between implicit attitudes and EDMU among unhappy adolescents. Implicit attitudes represent automatic, subconscious associations with digital media that create a tendency to perceive them positively, for example, as fun, enjoyable, or rewarding. In this context, impulsivity acts as a “process mechanism” that allows these implicit preferences to translate quickly into specific behaviors without conscious reflection or regulatory control. In other words, adolescents with positive implicit attitudes toward digital media are more prone to impulsive use, especially if they also exhibit weakened self-control.

These findings are consistent with previous research identifying impulsivity as a predictor of risky online behavior^39,92^ and low self-control as a vulnerability factor for various types of addictive behavior^44,69^. The combination of strong implicit motivations and weak self-regulation thus represents a significant risk for the development of EDMU. This mechanism appears particularly relevant for unhappy adolescents, where low self-control facilitates the translation of automatic tendencies into impulsive and potentially maladaptive digital behavior.

For explicit attitudes, however, the mediation by these self-regulatory factors was not significant, suggesting that explicit attitudes affect excessive digital media use more directly and independently of impulse regulation or self-control. This distinction underscores that automatic (implicit) and deliberate (explicit) attitudes may influence digital behavior through different mechanisms.

### Subjective happiness moderates the pathways linking attitudes to excessive digital media use

The results indicate that subjective happiness may shape the pathways through which attitudes translate into excessive digital media use among adolescents. Stratified models show that the influence of attitudes on excessive digital media use via impulsivity and self-control differs depending on the level of subjective happiness. The findings suggest that subjective happiness moderates how the pathways linking attitudes to excessive digital media use operate through impulsivity and self-control.

Although favorable attitudes toward social media were present in both groups, only unhappy adolescents engaged in ways that led to excessive use, and only in this group were implicit attitudes predictive of behavior. This pattern aligns with theoretical models of affective self-regulation^57,93^, which emphasize the role of the initial emotional state in influencing self-control and the balance between reflective and impulsive systems.

In contrast, happy adolescents appeared to be more resistant to these predictors. Their attitudes – whether explicit or implicit – did not lead to excessive use, and their stronger self-regulation mechanisms mitigated the impact of risky engagement. These results suggest that subjective happiness acts as a moderator of behavior in the digital domain. High levels of subjective happiness may protect adolescents from the influence of automatic preferences and reduce the emotional need for digital compensation^94^. Conversely, low subjective happiness may amplify the impact of impulsivity and reduce the effectiveness of conscious efforts to control use, as suggested by more recent models of affective regulation and media-related addiction^56,95^.

### Theoretical and practical implications

From a theoretical perspective, the findings suggest that the relationship between implicit and explicit attitudes toward social media and excessive digital media use should not be conceptualized as universal, but rather as contingent upon adolescents’ subjective well-being. The results are consistent with dual-process and self-regulation frameworks, indicating that automatic and reflective processes become particularly relevant when regulatory resources are compromised. Subjective happiness thus emerges as an important contextual moderator that may either attenuate or amplify the impact of attitudes on behavior. This implies that models of excessive digital media use should systematically integrate affective well-being as a central component, rather than treating it as a peripheral correlate.

From a clinical and applied standpoint, the findings support a differentiated approach to prevention and intervention. Adolescents with lower subjective happiness are more vulnerable to impulsive or excessive digital media use, particularly when positive attitudes toward digital media coincide with lower self-control. For this group, prevention efforts should extend beyond limiting screen time and include training in self-regulation, strategies to manage impulsivity, and guided awareness of how their attitudes influence their own digital behavior. Complementary support could involve offline activities that enhance adolescents’ subjective well-being, independent of attitudes toward digital media, and offer opportunities to practice social skills. In contrast, adolescents with higher subjective happiness generally exhibit lower risk of excessive use, and reinforcing self-regulatory skills may be sufficient as a universal preventive strategy. This graded approach enables educators and practitioners to adapt support to individual needs, making interventions more effective and targeted.

Future research should continue to examine how adolescents’ emotional experience (both the emotions they bring into interactions with digital media and those that arise as a result) interacts with implicit and explicit attitudes to shape digital behavior. Longitudinal and experimental designs could help clarify causal mechanisms and test interventions aimed at strengthening self-regulation, fostering awareness of how attitudes influence one’s own digital behavior, and promoting adaptive engagement with digital media, while also exploring how emotional experience influences both automatic and reflective evaluative processes.

#### Limitations

While the current findings provide a nuanced understanding of adolescent digital behavior, several limitations warrant consideration. First, the use of cross-sectional data precludes strong causal inference, and longitudinal or experimental designs are needed to clarify the directionality of effects. Although directional paths were specified based on theory, the design does not allow definitive conclusions about causal relationships. The observed associations may also reflect alternative directional patterns, including the possibility that excessive digital media use shapes attitudes, that impulsivity influences both attitudes and use, or that these variables operate in reciprocal and dynamically interrelated ways. Second, reliance on self-report measures may introduce bias due to social desirability or limited introspective accuracy. Although implicit attitude measures were included to mitigate this issue, they also have psychometric and interpretive limitations. Third, the study sample was drawn from a specific cultural and educational context, which may affect the generalizability of the results. Although moderation analyses were conducted to examine the role of subjective happiness, the cross-sectional design limits causal interpretation of these conditional effects. Lastly, excessive digital media use was assessed as a global construct, which does not allow differentiation between potentially distinct platform-specific or content-specific behavioral patterns. Despite these limitations, the study offers valuable insights into the interaction between attitudes, self-regulation, and adolescent digital behavior.

## Conclusion

This study advances understanding of adolescent digital media use by showing that implicit and explicit attitudes toward social media are linked to excessive use through distinct pathways. Among unhappy students, positive implicit attitudes operate both directly and indirectly via increased impulsivity and reduced self-control, whereas explicit attitudes exert only a direct effect. In happy adolescents, neither type of attitude significantly predicts excessive use, consistent with their lower levels of excessive engagement. Self-control and impulsivity emerge as central mechanisms translating implicit and explicit attitudes into actual behavior, shaping vulnerability to excessive digital media use. By distinguishing the differential roles of implicit and explicit attitudes and identifying how these pathways vary across adolescents, the findings provide a nuanced perspective on the psychological mechanisms underlying excessive digital media use.

## Data Availability

The datasets analyzed during the current study are available from the corresponding author on reasonable request.
